# 6-Gingerol Potentiates β-Cell Insulin Secretion Without Acting as an L-Cell GLP-1 Secretagogue

**DOI:** 10.3390/ijms27209114

**Published:** 2026-10-14

**Authors:** Vilasinee Intisang, Piyathida Yameephai, Kwanchanok Uppakara, Nutthapoom Pathomthongtaweechai

**Affiliations:** Chakri Naruebodindra Medical Institute, Faculty of Medicine Ramathibodi Hospital, Mahidol University, Samut Prakan 10540, Thailand; int.vilasinee@gmail.com (V.I.); piyatidayameepai@gmail.com (P.Y.); kwanchanok.upp@mahidol.ac.th (K.U.)

**Keywords:** 6-gingerol, GLP-1 receptor, insulin secretion, protein kinase A, enteroendocrine L-cells, pancreatic β-cells, incretin, type 2 diabetes

## Abstract

The incretin hormone glucagon-like peptide-1 (GLP-1) and its receptor (GLP-1R) are central regulators of glucose-stimulated insulin secretion and major targets in type 2 diabetes. 6-Gingerol, the principal bioactive constituent of ginger, has a reported insulinotropic action that depends on GLP-1 in vivo, but whether this reflects GLP-1 secretion from intestinal L-cells or signaling at the β-cell GLP-1R is unresolved. Using NCI-H716 and STC-1 L-cells and MIN6 β-cells, we examined whether 6-Gingerol acts as a GLP-1 secretagogue or at the β-cell. 6-Gingerol did not stimulate GLP-1 secretion. It had no effect in NCI-H716 cells and reduced secretion in STC-1 cells at concentrations that did not reduce viability. In MIN6 β-cells, 6-Gingerol potentiated insulin secretion in a bell-shaped manner. This effect was abolished by two mechanistically distinct GLP-1R antagonists and by protein kinase A inhibition and was accompanied by a GLP-1R-dependent rise in cAMP. MIN6 cells expressed *Glp1r* transcript, and Exendin-4 confirmed a functional receptor response. These findings suggest that 6-Gingerol acts at the β-cell through GLP-1R-, cAMP-, and PKA-dependent mechanisms and not as an L-cell GLP-1 secretagogue. At the pharmacological concentration used, these results suggest an in vitro mechanism rather than a physiological dietary effect, and in vivo validation is needed.

## 1. Introduction

Type 2 diabetes mellitus represents a growing global health challenge, driving demand for therapies that restore glucose-stimulated insulin secretion [1,2,3]. Among these, incretin-based therapies targeting the glucagon-like peptide-1 (GLP-1) axis have proven highly effective. GLP-1, secreted by intestinal L-cells, potentiates insulin secretion through the β-cell GLP-1 receptor (GLP-1R), a G_s_-coupled receptor, in a glucose-dependent manner, minimizing hypoglycemia risk, which is an advantage over glucose-independent insulin secretagogues and approaches that modulate insulin sensitivity or signaling without addressing insulin secretion itself. Receptor activation raises intracellular cAMP, which signals through both protein kinase A (PKA) and the exchange protein Epac2 to enhance insulin secretion [4]. GLP-1 receptor agonists have become a key component of contemporary type 2 diabetes management [5]. However, despite their clinical efficacy, their widespread use is often limited by high treatment costs and frequent gastrointestinal adverse effects, such as nausea and other upper gastrointestinal symptoms [6,7].

These challenges continue to drive the search for accessible, well-tolerated dietary phytochemicals capable of engaging the incretin axis. In principle, such compounds can act through two mechanistically distinct routes, either by stimulating endogenous GLP-1 secretion from intestinal L-cells, thereby acting as GLP-1 secretagogues, or by directly activating the GLP-1 receptor on pancreatic β-cells to stimulate insulin secretion [8,9,10]. Several phytochemicals, including berberine [11], resveratrol [12], and quercetin [13], have been reported to stimulate L-cell GLP-1 secretion through nutrient-sensing receptors on enteroendocrine cells. Whether these secretagogues also act directly on the β-cell GLP-1 receptor, however, has rarely been examined, leaving a key gap in the understanding of how phytochemicals engage this GLP-1 signaling axis.

6-Gingerol, the principal pungent constituent of ginger (*Zingiber officinale*), possesses well-documented anti-hyperglycemic properties [14]. These metabolic benefits have been attributed to classical pathways such as AMPK activation [15] and anti-inflammatory and antioxidant actions [16]. Importantly, Samad et al. demonstrated that the insulinotropic effect of 6-Gingerol in *db*/*db* mice requires GLP-1 and proceeds through the β-cell cAMP and PKA pathway and that 6-Gingerol raises plasma GLP-1 while lowering plasma DPP-4 [17]. However, because plasma GLP-1 reflects the balance of secretion and degradation across the whole animal, it remains unresolved whether 6-Gingerol acts as a direct L-cell GLP-1 secretagogue, acts at the β-cell GLP-1 receptor, or both.

Accordingly, the present study set out to dissect the cellular basis of the GLP-1-dependent insulinotropic action of 6-Gingerol. We hypothesized that 6-Gingerol acts at the β-cell GLP-1 receptor rather than as an intestinal L-cell secretagogue. Using intestinal L-cell (NCI-H716 and STC-1) and pancreatic β-cell (MIN6) models, we found that, contrary to the secretagogue paradigm, 6-Gingerol did not stimulate GLP-1 secretion from L-cells, yet it potentiated β-cell insulin secretion in a manner dependent on the GLP-1 receptor and PKA. These findings suggest that 6-Gingerol acts at the β-cell through a signaling cascade involving the GLP-1 receptor, cAMP, and PKA and not as a classical gut secretagogue. They raise the molecular question of whether the GLP-1 receptor signal arises from direct receptor engagement or from β-cell-intrinsic GLP-1.

## 2. Results

### 2.1. 6-Gingerol Preserves Cell Viability in Enteroendocrine and β-Cell Lines

**Figure 1:**
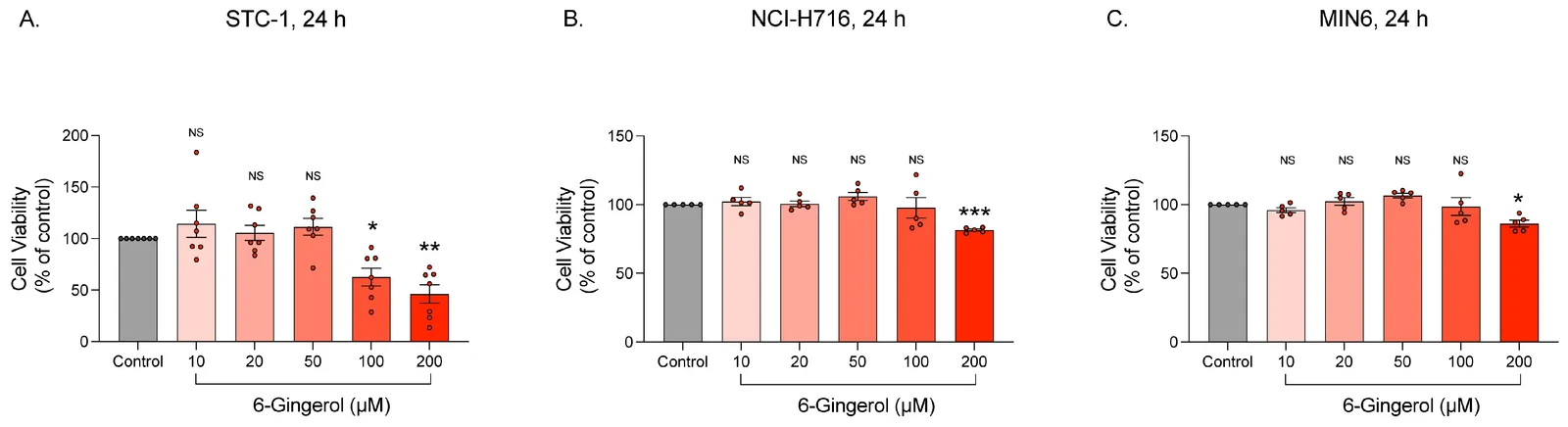
6-Gingerol preserves cell viability at working concentrations. Cell viability measured by MTT assay after 24 h of exposure to 6-Gingerol (10 to 200 µM) in (**A**) STC-1, (**B**) NCI-H716, and (**C**) MIN6 cells, expressed as a percentage of the within-experiment control (100%). Data are mean ± SEM (*n* = 7 for (**A**), *n* = 5 for (**B**), and *n* = 5 for (**C**)). Each concentration was compared against the normalized control (100%) using a one-sample *t*-test with Holm–Šídák correction across the concentrations in each panel. * *p* < 0.05, ** *p* < 0.01, *** *p* < 0.001 versus control; NS, not significant. Significance symbols indicate adjusted *p* values.

Cell viability was assessed by the MTT assay after a 24 h exposure to 6-Gingerol (10 to 200 µM) in murine STC-1 and human NCI-H716 L-cells and murine MIN6 β-cells (Figure 1). In all three lines, 6-Gingerol did not significantly affect viability at concentrations up to 50 µM. In STC-1 cells, viability was maintained at 10 to 50 µM (105.5 to 114.4% of control) but was reduced at 100 and 200 µM (62.7 ± 8.6%, *p* = 0.020; 46.3 ± 9.0%, *p* = 0.005) (Figure 1A). In NCI-H716 and MIN6 cells, viability was preserved up to 100 µM, with a modest reduction at 200 µM (NCI-H716, 81.5 ± 0.8%, *p* < 0.001; MIN6, 86.4 ± 2.5%, *p* = 0.028) (Figure 1B,C). Concentrations up to 50 µM were therefore non-cytotoxic in all three lines and were used as the working concentrations for the functional assays.

### 2.2. 6-Gingerol Potentiates Insulin Secretion in MIN6 β-Cells

**Figure 2:**
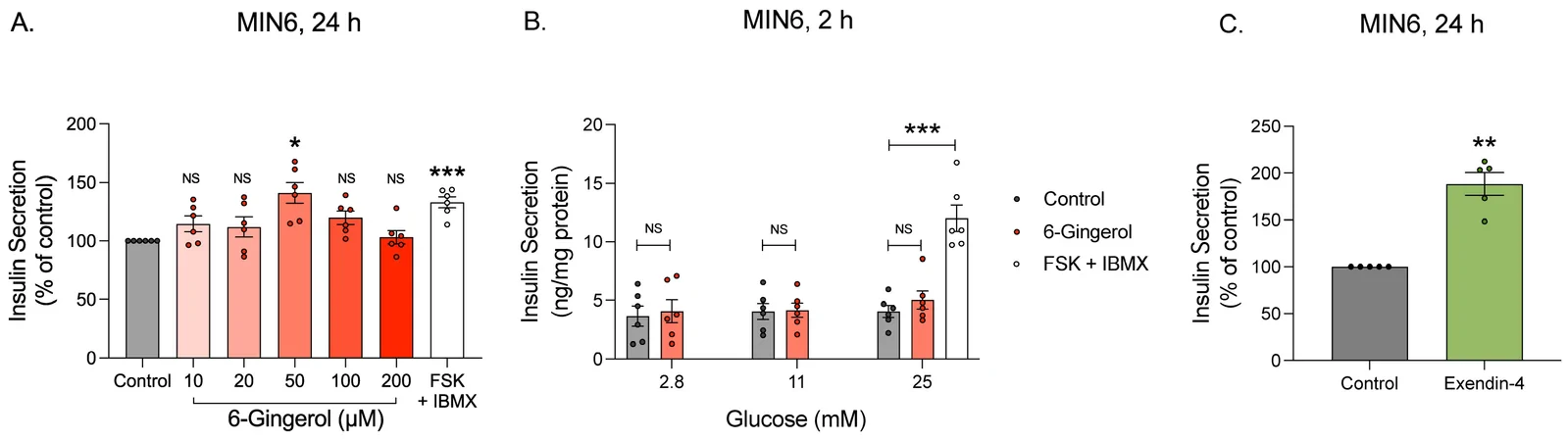
6-Gingerol enhances insulin secretion in MIN6 β-cells. (**A**) Insulin secretion measured by ELISA after 24 h of treatment with 6-Gingerol (10 to 200 µM), expressed as a percentage of control (100%). Forskolin (FSK, 10 µM) and 3-isobutyl-1-methylxanthine (IBMX, 10 µM) with an additional 15 mM glucose served as a positive control (*n* = 6). Data were analyzed by one-sample *t*-test versus 100% with Holm–Šídák correction across the five 6-Gingerol concentrations. The positive control was analyzed separately without correction. (**B**) Insulin secretion measured after pre-incubation in glucose-free KRBH buffer followed by 2 h of incubation with control or 6-Gingerol (50 µM) at 2.8, 11, or 25 mM glucose, normalized to total protein (ng/mg protein, *n* = 6). FSK + IBMX with an additional 15 mM glucose served as a positive control. Control and 6-Gingerol groups were analyzed by two-way repeated-measures ANOVA (both factors matched) with the Geisser–Greenhouse correction and Šídák’s multiple comparisons test comparing 6-Gingerol versus control at each glucose concentration (three comparisons). FSK + IBMX versus control at 25 mM glucose was analyzed separately by unpaired *t*-test with Welch’s correction. (**C**) Effect of the GLP-1 receptor agonist Exendin-4 (10 nM) on insulin secretion after 24 h, expressed as a percentage of control (*n* = 5). Data were analyzed by one-sample *t*-test versus 100%. Data are mean ± SEM. * *p* < 0.05, ** *p* < 0.01, *** *p* < 0.001 versus control (**A**,**C**) or versus control at 25 mM glucose (**B**). NS, not significant. Significance symbols indicate adjusted *p* values, except for FSK + IBMX in (**A**) and (**B**) and for Exendin-4 in (**C**), which were not adjusted.

MIN6 β-cells were treated with 6-Gingerol (10 to 200 µM) for 24 h (Figure 2A). 6-Gingerol produced a bell-shaped response, significantly increasing insulin secretion at 50 µM to 141.1 ± 9.0% of control (*p* = 0.029). Insulin concentrations were 6.54 ± 0.27 ng/mL under control conditions and 9.27 ± 0.80 ng/mL at 50 µM 6-Gingerol. Lower concentrations produced non-significant increases (114.6 ± 6.6% at 10 µM and 112.0 ± 8.5% at 20 µM), and the response returned toward baseline at higher concentrations (119.7 ± 5.7% at 100 µM, *p* = 0.070 and 103.2 ± 5.7% at 200 µM). The positive control forskolin and 3-isobutyl-1-methylxanthine (IBMX) increased secretion to 133.0 ± 4.7% (*p* = 0.0009, unadjusted). The loss of effect at higher concentrations occurred with preserved viability (Figure 1C), indicating a bell-shaped pharmacological response and not cytotoxicity. The 50 µM concentration was selected for the subsequent mechanistic experiments.

To confirm that this reflects insulin secretion and not accumulated insulin or changes in cell number, insulin secretion was measured after pre-incubation in glucose-free Krebs–Ringer bicarbonate HEPES (KRBH) buffer followed by a 2 h incubation at low (2.8 mM), intermediate (11 mM), and high (25 mM) glucose, with protein normalization (Figure 2B). Two-way repeated-measures ANOVA with the Geisser–Greenhouse correction showed no significant main effect of 6-Gingerol (*p* = 0.091) and no significant treatment × glucose interaction (*p* = 0.180). Post hoc comparisons were not significant at any glucose concentration (adjusted *p* = 0.520, 0.980 and 0.177 at 2.8, 11 and 25 mM, respectively). At 25 mM glucose, secretion was 5.02 ± 0.77 ng/mg protein with 6-Gingerol and 4.05 ± 0.52 ng/mg protein in control. Control cells showed no clear glucose response (3.66 ng/mg protein at 2.8 mM and 4.05 ng/mg protein at 25 mM, main effect of glucose *p* = 0.339), so this experiment could not establish whether the effect depends on glucose. The positive control forskolin and IBMX (with an additional 15 mM glucose) increased insulin secretion to 12.01 ± 1.13 ng/mg protein, compared with 4.05 ± 0.52 ng/mg protein in control at 25 mM glucose (*p* = 0.0004, Welch’s *t*-test, unadjusted).

To verify that MIN6 cells respond to a GLP-1 receptor agonist, cells were treated with Exendin-4 (10 nM), which increased insulin secretion to 188.4 ± 12.0% of control (*p* = 0.0018, unadjusted) (Figure 2C).

### 2.3. The Insulinotropic Effect Depends on the GLP-1 Receptor, cAMP, and PKA

**Figure 3:**
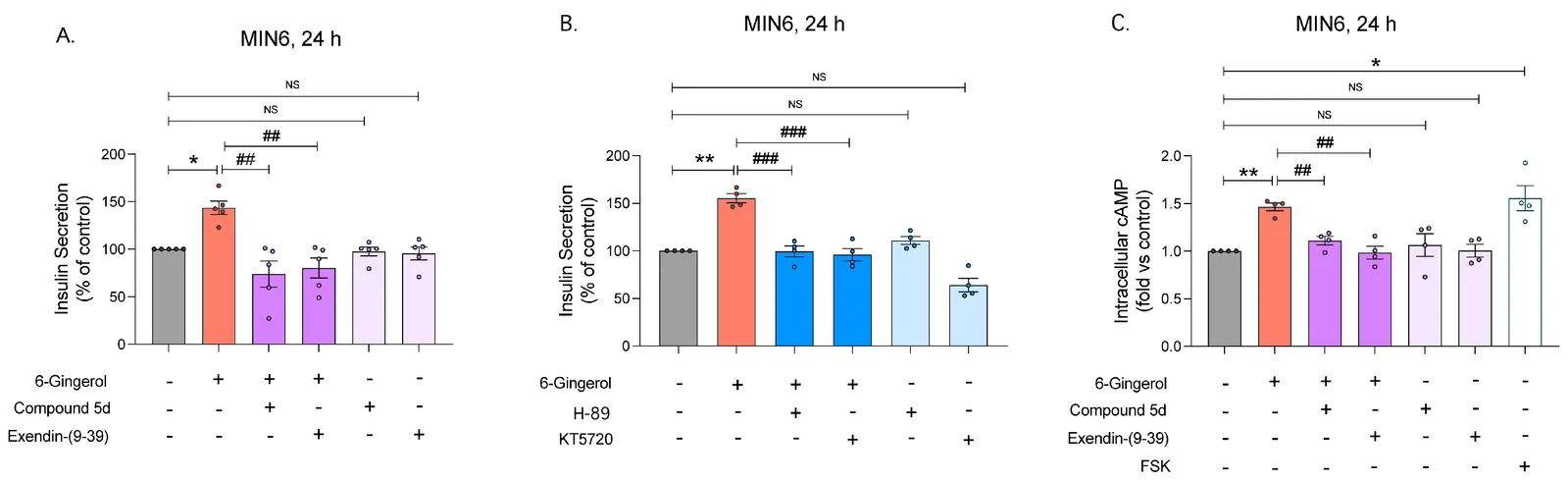
The insulinotropic effect of 6-Gingerol depends on GLP-1 receptor, cAMP, and PKA signaling in MIN6 β-cells. (**A**) Insulin secretion (percentage of control) by ELISA after 24 h of treatment with 6-Gingerol (50 µM) and the GLP-1 receptor antagonists compound 5d (100 nM) or Exendin-(9–39) (100 nM) (*n* = 5). (**B**) Insulin secretion after 24 h with 6-Gingerol (50 µM) and the PKA inhibitors H-89 (1 µM) or KT5720 (100 nM) (*n* = 4). (**C**) Intracellular cAMP (fold versus control) in cells treated with 6-Gingerol (50 µM) alone or with compound 5d (100 nM) or Exendin-(9–39) (100 nM). Forskolin (FSK, 20 µM) served as a positive control (*n* = 4). Data are mean ± SEM. Within each panel, the five groups were compared with control by one-sample *t*-test with Holm–Šídák correction. The combination groups were compared with 6-Gingerol by unpaired *t*-test with Welch’s correction and Holm–Šídák correction. FSK was analyzed separately without correction. * *p* < 0.05, ** *p* < 0.01 versus control; ## *p* < 0.01, ### *p* < 0.001 versus 6-Gingerol; NS, not significant. Significance symbols indicate adjusted *p* values, except for FSK in (**C**), which was not adjusted.

To test whether the GLP-1 receptor is involved, 6-Gingerol (50 µM) was co-administered with two mechanistically distinct GLP-1 receptor antagonists, the peptide antagonist Exendin-(9–39) (100 nM) and the small-molecule antagonist compound 5d (100 nM). 6-Gingerol increased insulin secretion to 143.6 ± 7.0% of control (*p* = 0.017), and both antagonists abolished this effect, with secretion not significantly differing from control (6-Gingerol + compound 5d, 73.9 ± 13.7%, *p* = 0.0041 versus 6-Gingerol and *p* = 0.428 versus control, 6-Gingerol + Exendin-(9–39), 80.2 ± 10.6%, *p* = 0.0032 versus 6-Gingerol and *p* = 0.428 versus control). Neither antagonist alone changed basal secretion (*p* = 0.798 for each) (Figure 3A).

Two structurally distinct PKA inhibitors, namely, H-89 (1 µM) and KT5720 (100 nM), abolished the effect (6-Gingerol + H-89, 99.5 ± 5.8%; 6-Gingerol + KT5720, 96.0 ± 6.4%; both *p* = 0.0008 versus 6-Gingerol and not different from control, *p* = 0.94 and *p* = 0.82, respectively) (Figure 3B). H-89 alone had no effect on basal secretion (110.9 ± 4.2%, NS), whereas KT5720 alone reduced it numerically (64.1 ± 7.2%) but not significantly after correction (*p* = 0.060), and an off-target effect cannot be excluded. Both inhibitors abolished the response, consistent with PKA dependence.

We next measured intracellular cAMP, which acts upstream of PKA (Figure 3C). 6-Gingerol increased cAMP to 1.47 ± 0.04-fold of control (*p* = 0.0075), which was abolished by both antagonists (6-Gingerol + compound 5d, 1.11 ± 0.04 fold, *p* = 0.0022 versus 6-Gingerol; 6-Gingerol + Exendin-(9–39), 0.99 ± 0.07 fold, *p* = 0.0022 versus 6-Gingerol), with forskolin (20 µM) as a positive control (1.56 ± 0.13 fold, *p* = 0.024, unadjusted). Together, dependence on the GLP-1 receptor, cAMP, and PKA is consistent with 6-Gingerol acting through a GLP-1 receptor–cAMP–PKA cascade.

### 2.4. 6-Gingerol Does Not Act as a GLP-1 Secretagogue in Intestinal L-Cells

**Figure 4:**
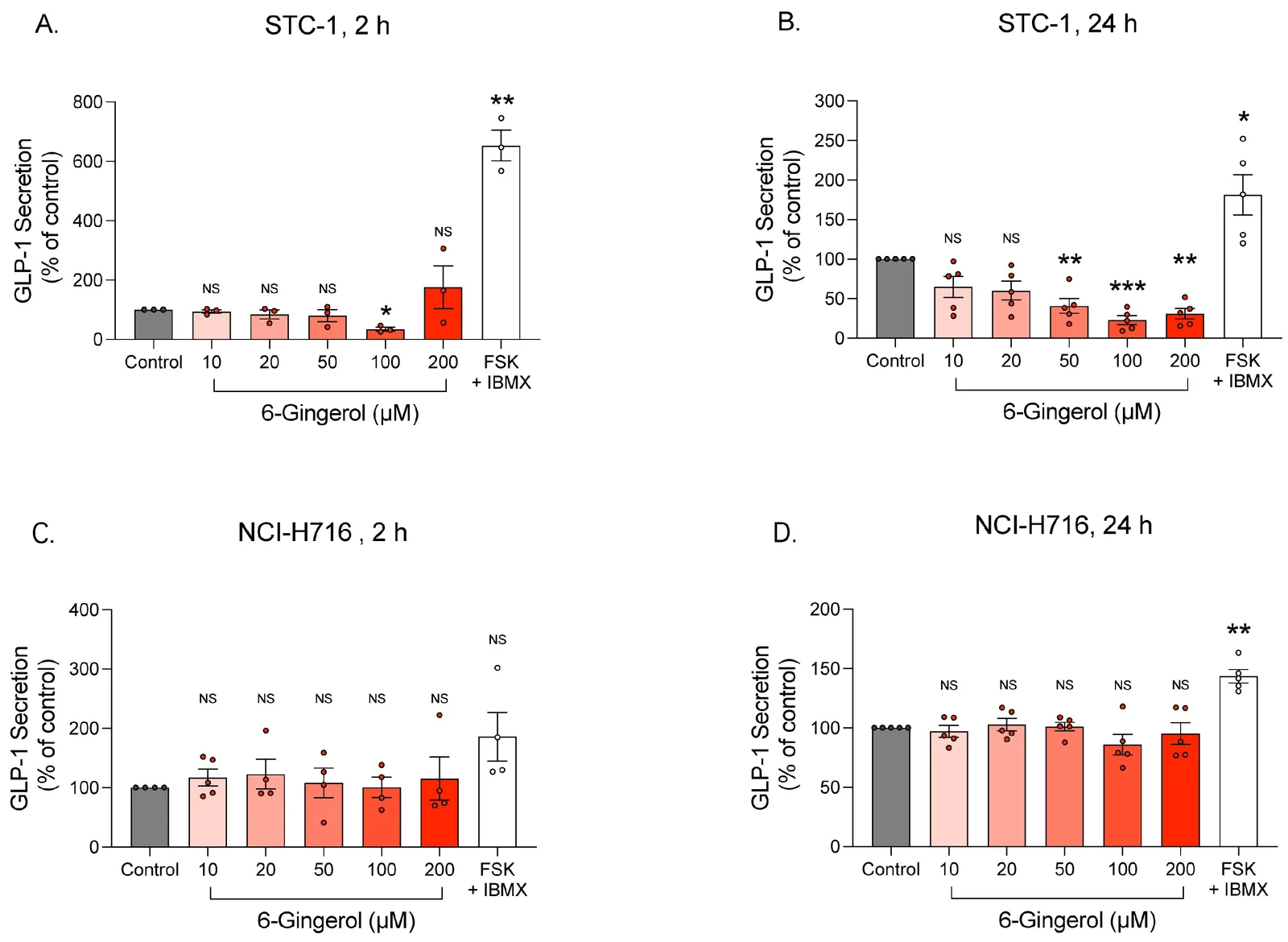
6-Gingerol does not act as a GLP-1 secretagogue in intestinal L-cells. GLP-1 secretion (percentage of control) by ELISA with the DPP-4 inhibitor teneligliptin (20 µM) present throughout in STC-1 cells at (**A**) 2 h and (**B**) 24 h, and NCI-H716 cells at (**C**) 2 h and (**D**) 24 h, treated with 6-Gingerol (10 to 200 µM). Forskolin (FSK) and IBMX served as a positive control. Data are mean ± SEM (*n* = 3 in (**A**), *n* = 5 in (**B**), *n* = 4 in (**C**) except *n* = 5 for 10 µM, and *n* = 5 in (**D**)). Data were analyzed by one-sample *t*-test versus 100% with Holm–Šídák correction across the five concentrations in each panel. FSK + IBMX was analyzed separately without correction. * *p* < 0.05, ** *p* < 0.01, *** *p* < 0.001 versus control; NS, not significant. Significance symbols indicate adjusted *p* values, except for FSK + IBMX.

GLP-1 secretion was measured at 2 h and 24 h in STC-1 and NCI-H716 cells, with the DPP-4 inhibitor teneligliptin (20 µM) present throughout to prevent degradation of secreted GLP-1 (Figure 4). In STC-1 cells, 6-Gingerol did not stimulate GLP-1 secretion at any concentration. Instead, secretion was reduced at 100 µM at 2 h (*p* = 0.043) (Figure 4A) and at 24 h at 50 µM and above (50 µM, *p* = 0.0098, 100 µM, *p* = 0.0008, 200 µM, *p* = 0.0019) (Figure 4B). At 50 µM, where viability was preserved, 6-Gingerol suppressed GLP-1 secretion independently of any effect on cell survival. At 100 and 200 µM, however, the larger reductions were accompanied by decreased viability, which may have contributed to the lower secretion. In NCI-H716 cells, 6-Gingerol did not change GLP-1 secretion at either timepoint (Figure 4C,D). Forskolin and IBMX significantly increased secretion at 2 h in STC-1 cells (653.3 ± 51.4% of control, *p* = 0.0085, unadjusted) and at 24 h in both cell lines (STC-1, 181.1 ± 25.4%, *p* = 0.033, NCI-H716, 143.4 ± 5.6%, *p* = 0.0015, unadjusted), confirming a functional secretory response. However, the response to forskolin and IBMX in NCI-H716 cells at 2 h did not reach significance (185.8 ± 40.9% of control, *p* = 0.127, unadjusted). These results indicate that 6-Gingerol does not act as a GLP-1 secretagogue in these cell models and that the increase in β-cell insulin secretion is not mediated by enhanced L-cell GLP-1 release.

### 2.5. 6-Gingerol Does Not Induce Autocrine GLP-1 Secretion from MIN6 β-Cells

**Figure 5:**
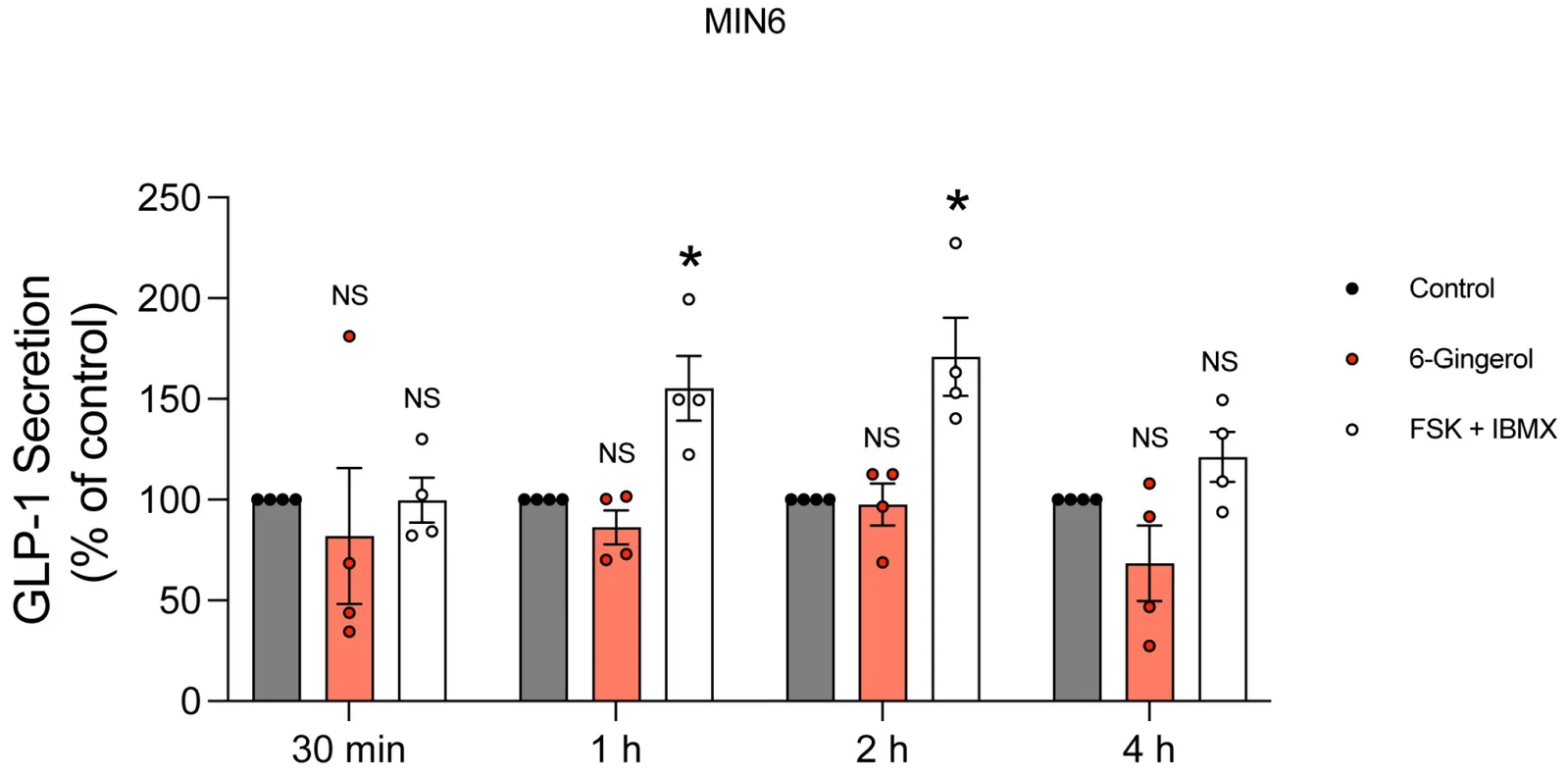
6-Gingerol does not stimulate GLP-1 release from MIN6 β-cells. GLP-1 release into the medium by ELISA at 30 min, 1 h, 2 h, and 4 h, expressed as a percentage of control, with the DPP-4 inhibitor teneligliptin (20 µM) present throughout. Cells were treated with control, 6-Gingerol (50 µM), or forskolin (FSK) and IBMX (positive control). Data are mean ± SEM (*n* = 4). 6-Gingerol was analyzed by one-sample *t*-test versus 100% with Holm–Šídák correction across the four timepoints. FSK + IBMX was analyzed separately without correction. * *p* < 0.05 versus control; NS, not significant. Significance symbols indicate adjusted *p* values, except for FSK + IBMX.

To test for a transient autocrine GLP-1 response, GLP-1 release from MIN6 cells was measured at 30 min, 1 h, 2 h, and 4 h with teneligliptin present throughout (Figure 5). 6-Gingerol did not significantly increase GLP-1 release at any of the tested timepoints. Forskolin and IBMX increased release at 1 h (155.3 ± 16.1% of control, *p* = 0.041, unadjusted) and 2 h (171.0 ± 19.4%, *p* = 0.035, unadjusted), although these did not remain significant after correction across the four timepoints (*p* = 0.13 for both). GLP-1 release returned toward baseline at 4 h (121.2 ± 12.4%). Because the response to cAMP stimulation was modest, the sensitivity of this assay may be limited. However, the absence of GLP-1 induction by 6-Gingerol across the full time course suggests that the GLP-1 receptor-dependent insulinotropic action does not appear to require an autocrine GLP-1 loop.

### 2.6. Expression of L-Cell and β-Cell Identity Genes and GLP-1R/GPR40 in STC-1, NCI-H716, and MIN6 Cells

**Figure 6:**
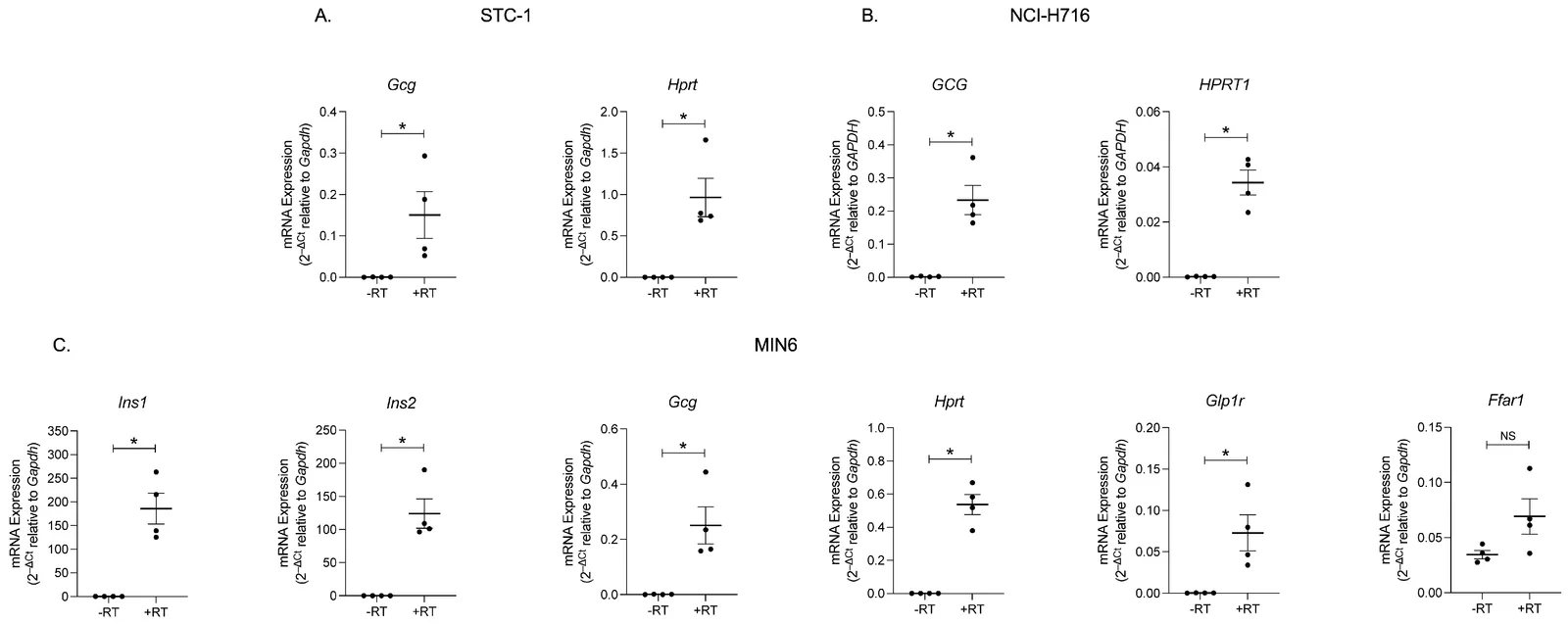
Expression of identity genes and GLP-1R/GPR40 in STC-1, NCI-H716, and MIN6 cells. (**A**) Relative mRNA expression (2^−ΔCt^, normalized to *Gapdh*) of *Gcg* and *Hprt* in STC-1 cells, comparing cDNA synthesized with (+RT) or without (−RT) reverse transcriptase as a background control. (**B**) Relative mRNA expression of *GCG* and *HPRT1* in NCI-H716 cells (+RT versus −RT). (**C**) Relative mRNA expression of *Ins1*, *Ins2*, *Gcg*, *Hprt*, *Glp1r*, and *Ffar1* in MIN6 cells (+RT versus −RT). Each point represents an independent biological replicate (*n* = 4). Bars show mean ± SEM. Comparisons between +RT and −RT were performed using the Mann–Whitney *U* test (two-tailed, without correction). * *p* < 0.05 versus −RT; NS, not significant. Relative mRNA expression was normalized to *Gapdh* in mouse cells and *GAPDH* in NCI-H716 cells.

To confirm the identity of the cell models and assess receptor expression relevant to the proposed mechanism, qPCR was performed for L-cell and β-cell identity genes, as well as *Glp1r* and *Ffar1* (GPR40), in STC-1, NCI-H716, and MIN6 cells (Figure 6).

In STC-1 cells, *Gcg* and *Hprt* were both significantly expressed above background (*p* = 0.0286 for each, Mann–Whitney *U* test; Figure 6A), confirming enteroendocrine L-cell identity. Similarly, in NCI-H716 cells, *GCG* and *HPRT1* were both significantly expressed above background (*p* = 0.0286 for each; Figure 6B).

In MIN6 cells, the β-cell markers *Ins1* and *Ins2*, together with *Gcg* and the housekeeping gene *Hprt*, were all significantly expressed above the −RT background (*p* = 0.0286 for each; Figure 6C). Notably, *Glp1r* was significantly expressed above background (*p* = 0.0286), whereas *Ffar1* expression did not significantly differ from background (*p* = 0.1143). These findings are consistent with a role for the GLP-1 receptor but do not exclude a contribution of GPR40 in mediating the insulinotropic effect of 6-Gingerol in MIN6 cells, because the sensitivity of qPCR does not exclude low-level *Ffar1* expression.

## 3. Discussion

The principal finding of this study is that 6-Gingerol potentiates insulin secretion in pancreatic β-cells through a GLP-1 receptor-, cAMP-, and PKA-dependent mechanism, without stimulating GLP-1 secretion from intestinal L-cells. In MIN6 β-cells, 6-Gingerol increased insulin secretion in a bell-shaped manner, and this effect was abolished by two mechanistically distinct GLP-1 receptor antagonists and by PKA inhibition. In contrast, 6-Gingerol did not stimulate GLP-1 secretion in human NCI-H716 cells but reduced GLP-1 secretion in murine STC-1 cells at 24 h, an effect that at 50 µM occurred without loss of viability. This distinction clarifies the cellular basis of the insulinotropic action of 6-Gingerol previously reported by Samad et al. [17].

A key contribution of the present study is the identification of the cellular source of the GLP-1 signal underlying the insulinotropic action of 6-Gingerol. Samad et al. showed in *db*/*db* mice that this action requires GLP-1 and proceeds through the β-cell cAMP and PKA pathway and reported a parallel rise in plasma GLP-1 together with reduced plasma DPP-4 activity [17]. Our β-cell data align with this cAMP/PKA-mediated pathway. The insulinotropic effect required the GLP-1 receptor, cAMP, and PKA, as it was abolished by two mechanistically distinct GLP-1 receptor antagonists, accompanied by a GLP-1 receptor-dependent rise in cAMP, and was blocked by two PKA inhibitors. We considered whether GPR40 (FFAR1), another β-cell receptor responsive to lipophilic ligands, might contribute. Although GPR40 can couple to G_s_/cAMP [18], our data suggest that the GLP-1 receptor is the principal mediator. First, both the insulinotropic and cAMP responses were abolished by GLP-1 receptor antagonists that do not act on GPR40. Second, under our assay conditions, *Ffar1* expression did not differ significantly from the −RT background, whereas *Glp1r* was detected above it (Figure 6). As qPCR detects transcript abundance rather than protein function or low-copy expression, this does not establish the complete absence of GPR40, and a minor contribution cannot be fully excluded. The use of separate L-cell and β-cell models helps distinguish whether the GLP-1 signal reflects secretion or reduced degradation, a distinction that circulating GLP-1 cannot provide. Direct measurement of GLP-1 in MIN6 supernatants showed that 6-Gingerol does not stimulate secretion from β-cells (Figure 5). Together with the absence of increased GLP-1 secretion from L-cells (Figure 4), this suggests that reduced DPP-4-mediated degradation, and not increased L-cell secretion, may account for the elevated plasma GLP-1 reported in vivo [17]. Samad et al. reported reduced plasma DPP-4 activity together with elevated plasma GLP-1 [17]. Because DPP-4 rapidly inactivates GLP-1, lower DPP-4 activity can raise circulating GLP-1 without any increase in L-cell secretion, which is consistent with our L-cell findings. 6-Gingerol therefore differs mechanistically from dietary phytochemicals characterized as L-cell GLP-1 secretagogues such as berberine [11], resveratrol [12], and quercetin [13] and from the AMPK-dependent [15] and anti-inflammatory [16] actions previously attributed to ginger constituents.

A central mechanistic question is how 6-Gingerol engages the GLP-1 receptor in a pure β-cell culture that lacks an intestinal source of GLP-1. Two non-exclusive possibilities exist. First, 6-Gingerol may engage the β-cell GLP-1 receptor directly, as an agonist or a positive allosteric modulator. Second, it may stimulate β-cell-intrinsic GLP-1 that acts in an autocrine or paracrine fashion. The second possibility is biologically plausible, as MIN6 cells express proglucagon and prohormone convertase 1/3 and possess a documented autocrine GLP-1 loop in which Exendin-4 amplifies and Exendin-(9–39) suppresses GLP-1 signaling [19]. In insulinoma β-cells, a GLP-1 receptor antagonist reduces insulin secretion by interrupting a functional loop with locally produced GLP-1 [20]. An autocrine loop requires GLP-1 to be secreted before acting on the receptor. The absence of any increase in GLP-1 release at any timepoint across the full time course is therefore inconsistent with a secreted intermediate and suggests direct engagement of the β-cell receptor. We frame this conclusion conservatively as dependence on the GLP-1 receptor rather than direct agonism. As a small phenolic molecule lacking structural homology to the GLP-1 peptide, 6-Gingerol is unlikely to engage the orthosteric peptide-binding site directly and may instead act through allosteric or indirect enhancement of GLP-1 receptor signaling, a distinction that remains to be resolved in a heterologous GLP-1 receptor-expressing system. The use of two antagonists with different mechanisms of action further supports receptor dependence. Exendin-(9–39) is a peptide antagonist that competes at the orthosteric GLP-1-binding site, whereas compound 5d acts non-competitively at a distinct site [21]. Blockade at either site abolished the response, indicating that the insulinotropic effect requires a functional GLP-1 receptor and is unlikely to reflect an off-target action of either antagonist alone.

The PKA dependence of the response further constrains the underlying mechanism. It localizes the signal to the cAMP and PKA arm canonically engaged by the G_s_-coupled GLP-1 receptor. cAMP also signals through Epac2 in parallel with PKA in β-cells [4]. The near-complete blockade by both H-89 and KT5720 is consistent with PKA being the main effector here, although an Epac2 contribution cannot be excluded.

The intestinal L-cell results showed a different pattern. 6-Gingerol did not affect GLP-1 secretion in human NCI-H716 cells but markedly reduced secretion in murine STC-1 cells at 24 h. At 50 µM, this reduction occurred without loss of viability and therefore reflects suppression of secretion and not cytotoxicity, whereas the further reduction at 100 and 200 µM was accompanied by reduced STC-1 viability and may partly reflect cytotoxicity, not suppressed secretion alone. This in vitro result differs from the elevated plasma GLP-1 reported in vivo, but the two remain consistent, as the in vivo increase may largely reflect reduced DPP-4 activity, not enhanced L-cell secretion. STC-1 is a mixed enteroendocrine line with high constitutive secretory tone, and the suppression may reflect a reduction of this constitutive output. Its mechanism is beyond the scope of the present study and should be interpreted with caution. The two L-cell lines responded differently. 6-Gingerol had no effect in human NCI-H716 cells but reduced secretion in mouse STC-1 cells. This may reflect a species difference or a property of each cell line. The reduction in STC-1 cells at 50 µM appeared only after 24 h, so an indirect effect of the long treatment cannot be excluded. A shorter exposure, such as 4 h, was not tested. In addition, the response to forskolin and IBMX in NCI-H716 cells at 2 h was variable (Figure 4C), so that timepoint is less informative. Nevertheless, 6-Gingerol did not increase GLP-1 secretion in either line, so there is no evidence that it acts as an L-cell secretagogue in these models.

Several limitations should be acknowledged. First, the effective concentration (50 µM) substantially exceeds concentrations attainable in vivo. Free 6-Gingerol is generally undetectable in human plasma after oral ginger administration, with only glucuronide and sulfate conjugates detected even after doses of up to 2 g [22]. Reported plasma C_max_ values for 6-Gingerol and its metabolites in humans are in the sub-micromolar to low-micromolar range [23], and its absolute oral bioavailability is below 2% in rodent studies [24]. Because β-cells are exposed to systemic concentrations, not the higher luminal levels at the gut surface, the 50 µM concentration used here represents a pharmacological, proof-of-mechanism condition and not a physiologically attainable dose. Effective β-cell exposure in vivo would likely require formulations with enhanced bioavailability or targeted delivery. The bell-shaped manner, with loss of activity at higher non-cytotoxic concentrations, is consistent with a receptor-mediated effect. Such biphasic, inverted-U relationships are well documented for phenolic and polyphenolic compounds, in which low-to-moderate concentrations produce a modest stimulatory effect while higher concentrations attenuate or reverse it [25].

Second, this study used immortalized cell lines, and validation in primary islets, primary L-cells, and in vivo models is warranted. The suggestion that reduced DPP-4-mediated degradation contributes to elevated plasma GLP-1 in vivo also remains a hypothesis requiring direct testing.

Third, the mechanistic evidence has limits. Both PKA inhibitors have recognized off-target activity. H-89 has been reported to inhibit several other kinases, and KT5720 alone reduced mean basal secretion, although not significantly after correction. PKA activity was not measured directly, and an Epac2 inhibitor was not tested, so the PKA dependence should be interpreted with caution.

Fourth, the expression and autocrine data are also limited. GLP-1 receptor expression was shown only as detection above the −RT background, without a positive control cell line or protein-level confirmation. Because of the sensitivity limit of qPCR, the lack of a significant *Ffar1* signal above background does not establish the absence of GPR40. In addition, forskolin and IBMX increased GLP-1 release in MIN6 cells at 1 and 2 h but not at 4 h, and this increase did not remain significant after correction. It may reflect degradation of GLP-1 in the medium or feedback regulation, and it limits the sensitivity of the autocrine experiment.

Finally, the sample sizes were modest (*n* = 4 to 5 in Figure 3), which limits the power to detect small effects such as that of KT5720 alone. In the 2 h insulin secretion assay, control cells showed no clear response to glucose, and 6-Gingerol had no significant effect at any glucose concentration, so this study cannot determine whether the effect depends on glucose concentration. This question requires a model with a robust glucose-stimulated insulin secretion response, such as primary islets.

Future studies using GLP-1 receptor-overexpressing or -deficient cells, receptor-binding assays, and CRE-luciferase reporters would distinguish direct agonism, positive allosteric modulation, and enhancement of endogenous signaling, complemented by confirmation of GLP-1 receptor and GPR40 expression in the L-cell lines used. In vivo validation would further establish physiological relevance. In summary, these in vitro data suggest that 6-Gingerol acts at the β-cell through the GLP-1 receptor–cAMP–PKA pathway, without acting as a GLP-1 secretagogue.

## 4. Materials and Methods

### 4.1. Cell Culture

The murine enteroendocrine L-cell line STC-1 (ATCC; Manassas, VA, USA) was cultured in Dulbecco’s Modified Eagle’s Medium (DMEM) supplemented with 10% heat-inactivated fetal bovine serum (FBS; Thermo Fisher Scientific, Waltham, MA, USA) and penicillin (100 U/mL) and streptomycin (100 µg/mL) (Cat. No. 15140122, Gibco, Grand Island, NY, USA). The human enteroendocrine L-cell line NCI-H716 (ATCC; CCL-251, Manassas, VA, USA) was cultured in RPMI-1640 (Cat. No. 820206-1, BIOMEDIA, Nonthaburi, Thailand) supplemented with 10% heat-inactivated FBS and the same antibiotics. For functional assays, NCI-H716 cells were plated on Matrigel-coated plates (Corning, NY, USA) to promote adherence. The murine pancreatic β-cell line MIN6 (ATCC; Manassas, VA, USA) was cultured in DMEM containing 4500 mg/L glucose, supplemented with 10% heat-inactivated FBS and 1% penicillin–streptomycin, and was used between passages 30 and 45. All lines were maintained at 37 °C in a humidified atmosphere of 95% air and 5% CO_2_, with medium renewed every 2 days and subcultured at 70 to 90% confluency using 0.25% trypsin–EDTA (Cat. No. 25200072, Gibco, Paisley, UK).

### 4.2. Reagents

6-Gingerol (Sigma-Aldrich, St. Louis, MO, USA) was dissolved in DMSO and diluted in serum-free medium, with the final vehicle concentration kept below 0.1%, and control cells received the same concentration of DMSO. MTT and DMSO were from Sigma-Aldrich (St. Louis, MO, USA) and Invitrogen (Eugene, OR, USA), respectively. Two mechanistically distinct GLP-1 receptor antagonists were used, Exendin-(9–39) (Bio-Techne, Minneapolis, MN, USA) and the non-competitive antagonist compound 5d (GLP-1R antagonist 1; cat. no. HY-101116, MedChemExpress, Monmouth Junction, NJ, USA). Two structurally distinct PKA inhibitors, namely, H-89 dihydrochloride and KT5720 (cat. no. HY-N6789), the DPP-4 inhibitor teneligliptin hydrobromide hydrate (cat. no. HY-14806B), and the GLP-1 receptor agonist Exendin-4 (cat. no. HY-13443) were from MedChemExpress (Monmouth Junction, NJ, USA). Exendin-4 was used as a positive control for GLP-1 receptor signaling. Forskolin and IBMX (both Sigma-Aldrich, St. Louis, MO, USA) were used as positive controls.

### 4.3. MTT Cell Viability Assay

STC-1 (5 × 10^4^ cells/well), NCI-H716 (4 × 10^4^ cells/well), and MIN6 (5 × 10^4^ cells/well) cells were plated in 96-well plates. At approximately 80% confluency, cells were treated with 6-Gingerol (10 to 200 µM) in serum-free medium for 24 h. The treatment medium was then removed, and freshly prepared MTT solution (0.5 mg/mL in serum-free medium, 100 µL/well) was added. After incubation at 37 °C for 2 h, the formazan was dissolved in 100 µL of DMSO. The absorbance was read at 570 nm (BioTek Synergy Neo2, Agilent, Santa Clara, CA, USA).

### 4.4. GLP-1 Secretion Assay

STC-1 and NCI-H716 cells (1.5 × 10^5^ cells/well) were plated in 24-well plates. At approximately 80% confluency, the cells were treated with 6-Gingerol (10 to 200 µM) for 2 and 24 h in the presence of the DPP-4 inhibitor teneligliptin (20 µM) to prevent degradation of secreted GLP-1. Forskolin (10 µM) and IBMX (10 µM) served as a positive control. For the MIN6 GLP-1 experiment, MIN6 cells were treated with 6-Gingerol (50 µM) or forskolin and IBMX, and the supernatant was collected at 30 min, 1 h, 2 h, and 4 h. Teneligliptin (20 µM) was present throughout. Secreted active GLP-1 was quantified using a GLP-1 ELISA kit (Merck Millipore, Burlington, MA, USA) according to the manufacturer’s instructions, with the fluorescence read at excitation 355 nm and emission 460 nm using a BioTek Synergy Neo2 microplate reader (Agilent, Santa Clara, CA, USA).

### 4.5. Insulin Secretion Assay

MIN6 cells were seeded in 24-well plates at 3 × 10^5^ cells/well and cultured for 24 h. All 24 h treatments were performed in DMEM containing 4500 mg/L (25 mM) glucose. For the concentration response, cells were treated with 6-Gingerol (10 to 200 µM) for 24 h. For the mechanistic experiments, cells were treated with 6-Gingerol (50 µM) alone or were co-treated with a GLP-1 receptor antagonist (Exendin-(9–39), 100 nM, or compound 5d, 100 nM)**,** a PKA inhibitor (H-89, 1 µM, or KT5720, 100 nM), or the GLP-1 receptor agonist Exendin-4 (10 nM), for 24 h. An additional 15 mM glucose together with forskolin (10 µM) and IBMX (10 µM) served as a positive control. For the acute insulin secretion assay, cells were washed and pre-incubated in glucose-free KRBH buffer for 20 min and then were incubated for 2 h with vehicle control or 6-Gingerol (50 µM) in glucose-free DMEM (Gibco, Paisley, UK) containing 2.8, 11, or 25 mM glucose. Forskolin (10 µM) and IBMX (10 µM) with an additional 15 mM glucose served as a positive control. Secreted insulin was normalized to total cellular protein (ng/mg protein) determined by the Bradford assay (Cat. No. 23200, Thermo Scientific, Rockford, IL, USA). The supernatant was collected, and secreted insulin was quantified using a Rat/Mouse Insulin ELISA kit (cat. no. EZRMI-13K, Merck KGaA, Darmstadt, Germany), with absorbance read at 450 and 590 nm using a BioTek Synergy Neo2 microplate reader.

### 4.6. Intracellular cAMP Measurement

Intracellular cAMP was measured using the cAMP Parameter Assay Kit (KGE002B, R&D Systems, Minneapolis, MN, USA). MIN6 cells were seeded at 1.5 × 10^5^ cells/well in 24-well plates and then were treated with 6-Gingerol (50 µM) alone or co-treated with a GLP-1 receptor antagonist (Exendin-(9–39), 100 nM, or compound 5d, 100 nM) for 24 h. Forskolin (20 µM) treatment for 15 min served as a positive control. Cells were collected, centrifuged at 1300× *g* at 4 °C for 10 min, washed with ice-cold PBS, and lysed. Lysates were subjected to two to three freeze-thaw cycles at −80 °C and centrifuged at 12,000× *g* at 4 °C for 20 min. Total protein was determined by the Bradford assay (Thermo Scientific, Rockford, IL, USA). cAMP was quantified per the manufacturer’s instructions (absorbance 450 nm, background correction 570 nm) using a BioTek Synergy Neo2 microplate reader and expressed relative to control.

### 4.7. Quantitative RT-PCR

Total RNA was extracted from STC-1, NCI-H716, and MIN6 cells using the Monarch Total RNA Miniprep Kit (#T2110, New England Biolabs, Ipswich, MA, USA). The RNA concentration and purity were assessed by NanoPhotometer NP80 Touch (Implen GmbH, Munich, Germany), and samples with a 260/280 ratio of 2.0 or greater were used for downstream analysis. Complementary DNA (cDNA) was synthesized from 120 ng of total RNA using iScript Reverse Transcription Supermix for RT-qPCR (Cat. No. 1708841, Bio-Rad Laboratories, Hercules, CA, USA). For each sample, a parallel reaction lacking reverse transcriptase (−RT) was prepared to control for genomic DNA contamination.

Quantitative PCR was performed using iTaq Universal SYBR Green Supermix (Cat. No. 1725121, Bio-Rad Laboratories, Hercules, CA, USA) on a CFX96 Touch Real-Time PCR Detection System (Bio-Rad Laboratories, Hercules, CA, USA), with primers targeting *Gcg* and *Hprt* in STC-1 cells, *GCG* and *HPRT1* in NCI-H716 cells, and *Ins1*, *Ins2*, *Gcg*, *Hprt*, *Glp1r*, and *Ffar1* in MIN6 cells (primer sequences listed in Supplementary Table S1). Cycling conditions were 95 °C for 2 min, followed by 40 cycles of 95 °C for 5 s and 60 °C for 30 s. Melt curve analysis was performed to confirm primer specificity. Data were analyzed using CFX Maestro Software (version 2.2, Bio-Rad Laboratories, Hercules, CA, USA). Experiments were performed with *n* = 4 independent biological replicates (separate cell passages). Relative gene expression was calculated using the 2^−ΔCt^ method, normalized to the housekeeping gene *Gapdh* in STC-1 and MIN6 cells and *GAPDH* in NCI-H716 cells.

### 4.8. Statistical Analysis

Data are presented as the mean ± SEM from independent biological replicates. Normality was assessed by the Shapiro–Wilk test. Data were normalized to the within-experiment control (100%, or 1.0-fold for cAMP). As the normalized control has zero variance, comparisons against control used one-sample *t*-tests with Holm–Šídák correction across concentrations or timepoints. Unless stated otherwise, *p* values are Holm–Šídák adjusted. Comparisons between treatment groups used unpaired *t*-tests with Welch’s correction, and Holm–Šídák correction was applied within each family of comparisons as specified in the figure legends. Insulin secretion at different glucose concentrations (Figure 2B) was analyzed by two-way repeated-measures ANOVA using the Geisser–Greenhouse correction followed by Šídák’s multiple comparisons test. Positive controls were analyzed separately without correction. For qRT-PCR, expression in cDNA (+RT) samples was compared with the corresponding −RT control using the Mann–Whitney *U* test (two-tailed, without correction), as −RT values were near the assay’s detection limit with minimal variance. Outliers were identified using the ROUT method (Q = 1%). Analyses used GraphPad Prism 10.4.2 (GraphPad Software, Boston, MA, USA). A value of *p* < 0.05 was considered significant.

## 5. Conclusions

In conclusion, our data suggest that 6-Gingerol potentiates β-cell insulin secretion through a mechanism dependent on the GLP-1 receptor and PKA, without acting as an L-cell GLP-1 secretagogue. By assessing L-cells and β-cells separately, this study suggests that 6-Gingerol exerts its GLP-1-dependent insulinotropic action through the β-cell GLP-1 receptor pathway and that the rise in circulating GLP-1 reported in vivo need not reflect increased L-cell secretion. These findings propose an in vitro mechanism, observed at a pharmacological concentration, in which 6-Gingerol acts at the β-cell GLP-1 receptor pathway rather than at the point of hormone secretion. In vivo validation is needed, together with protein-level confirmation of GLP-1 receptor expression, which was shown here at the transcript level only, and experiments that distinguish direct receptor engagement from β-cell-derived GLP-1.

## Data Availability

The data presented in this study are available on request from the corresponding author.

## Supplementary Materials

The following supporting information can be downloaded at: https://www.mdpi.com/article/10.3390/ijms27209114/s1.

## Abbreviations

The following abbreviations are used in this manuscript:

AMPK	AMP-activated protein kinase
ANOVA	Analysis of variance
cAMP	Cyclic adenosine monophosphate
C_max_	Maximum plasma concentration
DMEM	Dulbecco’s Modified Eagle’s Medium
DMSO	Dimethyl sulfoxide
DPP-4	Dipeptidyl peptidase-4
ELISA	Enzyme-linked immunosorbent assay
FBS	Fetal bovine serum
FSK	Forskolin
GLP-1	Glucagon-like peptide-1
GLP-1R	Glucagon-like peptide-1 receptor
GPR40	G protein-coupled receptor 40
G_q_	Q-class G protein
G_s_	Stimulatory G protein
IBMX	3-Isobutyl-1-methylxanthine
KRBH	Krebs–Ringer Bicarbonate HEPES
MTT	3-(4,5-Dimethylthiazol-2-yl)-2,5-diphenyltetrazolium bromide
PKA	Protein kinase A
SEM	Standard error of the mean
