# Supplementary material for: 6-Gingerol Potentiates β-Cell Insulin Secretion Without Acting as an L-Cell GLP-1 Secretagogue
Source: Int J Mol Sci. 2026 Oct 14;27(20):9114. doi: 10.3390/ijms27209114 (PMC13704961; doi:10.3390/ijms27209114)
Supplement: Supplementary file 1 [file ijms-27-09114-s001.zip › ijms-4437911-supplementary.pdf]

Supplementary Table S1. Primer sequences used for quantitative RT-PCR.

Cell line	Species	Gene	Accession no.	Forward primer (5′→3′)	Reverse primer (5′→3′)
STC-1	Mouse	*Gcg*	NM_008100	ATCTTGCCACCAGGGACTTC	AAGTGACTGGCACGAGATGT
STC-1	Mouse	*Hprt*	NM_013556	CTGGTGAAAAGGACCTCTCGAAG	CCAGTTTCACTAATGACACAAACG
STC-1	Mouse	*Gapdh*	NM_008084	CATCACTGCCACCCAGAAGACTG	ATGCCAGTGAGCTTCCCGTTCAG
NCI-H716	Human	*GCG*	NM_002054	TGGCAACGTTCCCTTCAAGA	GATCACTGAGTGGGTCTGCC
NCI-H716	Human	*HPRT1*	NM_000194	CATTATGCTGAGGATTTGGAAAGG	CTTGAGCACACAGAGGGCTACA
NCI-H716	Human	*GAPDH*	NM_002046	GTCTCCTCTGACTTCAACAGCG	ACCACCCTGTTGCTGTAGCCAA
MIN6	Mouse	*Ins1*	NM_008386	CGTGGCTTCTTCTACACACCCA	TGCAGCACTGATCCACAATGCC
MIN6	Mouse	*Ins2*	NM_008387	CGTGGCTTCTTCTACACACCCA	TCCAGTGCCAAGGTCTGAAGGT
MIN6	Mouse	*Gcg*	NM_008100	ATCTTGCCACCAGGGACTTC	AAGTGACTGGCACGAGATGT
MIN6	Mouse	*Hprt*	NM_013556	CTGGTGAAAAGGACCTCTCGAAG	CCAGTTTCACTAATGACACAAACG
MIN6	Mouse	*Glp1r*	NM_021332	CATCGTGGTATCCAAACTGA	GCTCGTCCATCACAAAGG
MIN6	Mouse	*Ffar1*	NM_194057	CCATTCTGCTCTTCTTTCTG	GGGTTTATGAAACTAGCCAC
MIN6	Mouse	*Gapdh*	NM_008084	CATCACTGCCACCCAGAAGACTG	ATGCCAGTGAGCTTCCCGTTCAG
